# The German Quality Network Sepsis: study protocol for the evaluation of a quality collaborative on decreasing sepsis-related mortality in a quasi-experimental difference-in-differences design

**DOI:** 10.1186/s13012-017-0706-5

**Published:** 2018-01-18

**Authors:** Daniel Schwarzkopf, Hendrik Rüddel, Matthias Gründling, Christian Putensen, Konrad Reinhart

**Affiliations:** 1 Integrated Research and Treatment Center for Sepsis Control and Care (CSCC), Jena University Hospital, Am Klinikum 1, 07747 Jena, Germany; 2 Department for Anesthesiology and Intensive Care Medicine, Jena University Hospital, Am Klinikum 1, 07747 Jena, Germany; 3 Department of Anesthesiology and Intensive Care Medicine, Ernst-Moritz-Arndt-University, Sauerbruchstraße, 17475 Greifswald, Germany; 4 Department of Anesthesiology and Intensive Care Medicine, University of Bonn, Sigmund-Freud-Str. 25, 53105 Bonn, Germany

**Keywords:** Sepsis, Mortality, Quality improvement, Hospitals, Risk adjustment, Administrative claims, Interdisciplinary health team

## Abstract

**Background:**

While sepsis-related mortality decreased substantially in other developed countries, mortality of severe sepsis remained as high as 44% in Germany. A recent German cluster randomized trial was not able to improve guideline adherence and decrease sepsis-related mortality within the participating hospitals, partly based on lacking support by hospital management and lacking resources for documentation of prospective data. Thus, more pragmatic approaches are needed to improve quality of sepsis care in Germany. The primary objective of the study is to decrease sepsis-related hospital mortality within a quality collaborative relying on claims data.

**Method:**

The German Quality Network Sepsis (GQNS) is a quality collaborative involving 75 hospitals. This study protocol describes the conduction and evaluation of the start-up period of the GQNS running from March 2016 to August 2018. Democratic structures assure participatory action, a study coordination bureau provides central support and resources, and local interdisciplinary quality improvement teams implement changes within the participating hospitals. Quarterly quality reports focusing on risk-adjusted hospital mortality in cases with sepsis based on claims data are provided. Hospitals committed to publish their individual risk-adjusted mortality compared to the German average. A complex risk-model is used to control for differences in patient-related risk factors. Hospitals are encouraged to implement a bundle of interventions, e.g., interdisciplinary case analyses, external peer-reviews, hospital-wide staff education, and implementation of rapid response teams. The effectiveness of the GQNS is evaluated in a quasi-experimental difference-in-differences design by comparing the change of hospital mortality of cases with sepsis with organ dysfunction from a retrospective baseline period (January 2014 to December 2015) and the intervention period (April 2016 to March 2018) between the participating hospitals and all other German hospitals. Structural and process quality indicators of sepsis care as well as efforts for quality improvement are monitored regularly.

**Discussion:**

The GQNS is a large-scale quality collaborative using a pragmatic approach based on claims data. A complex risk-adjustment model allows valid quality comparisons between hospitals and with the German average. If this study finds the approach to be useful for improving quality of sepsis care, it may also be applied to other diseases.

**Trial registration:**

ClinicalTrials.gov NCT02820675.

## Background

Sepsis is one of the major challenges for health care today [1]. It is the leading cause of death among infectious diseases [2] and might also be the leading cause of preventable deaths in hospitals [3, 4]. With estimated yearly costs in the USA and Germany of 27 billion USD and 7.7 Euro respectively, sepsis is one of the most expensive inpatient diseases [5, 6]. Recognizing severe shortcomings in prevention, diagnosis, and treatment of sepsis, the World Health Organization adopted a resolution in May 2017, which urges all member states to invest in improvement of sepsis care [7].

Guidelines for treating sepsis recommend early recognition by standardized screening, early administration of antibiotics, early adequate fluid resuscitation, and source control [8]. Several studies have shown positive effects of early therapy on patients’ outcomes, but at the same time guideline adherence is often low [3, 4, 9–16]. By using multifaceted interventions to increase guideline adherence on early adequate diagnosis and therapy of sepsis, several regional, national, and international quality initiatives were able to substantially reduce sepsis mortality in the participating hospitals [17–23].

In 2013, 279.530 patients were diagnosed with sepsis, severe sepsis, or septic shock in German hospitals. Comparable to other high-income countries, sepsis incidence had increased from 2007 to 2013 by 5.7% per year [5, 24]. Hospital mortality among patients with severe sepsis in Australia, England, and the USA decreased from 35 to 18.5%, from 45.5 to 32.1%, and from 37 to 29%, respectively during the 2000s [25–27]. In Germany, it decreased by only 4.2% from 47.8 to 43.6% between 2003 and 2013 [24, 28].

The MEDUSA trial (Medical EDUcation for sepsis Source control and Antibiotics, ClinicalTrials.gov identifier NCT01187134) was the first multi-center quality initiative for reducing mortality among inpatient cases with severe sepsis in Germany [12, 29]. This cluster randomized controlled trial involving 42 hospitals used a quality reporting based on prospectively gathered clinical data. A bottom-up approach led by the local intensive care departments was used to implement a multifaceted intervention within a 2-year intervention period. The intervention led to no substantial improvement in guideline adherence and no decrease in 28-day mortality [29]. Expert interviews and surveys of local quality improvement teams identified lacking support by hospital management, lacking support of the project by other departments, and lacking time for data documentation and implementation of interventions as major barriers to change [30]. Incomplete inclusion of cases with sepsis and delayed documentation led to decreased validity of reporting of quality indicators and reduced power to detect effects of the intervention. Major lessons drawn from these experiences are as follows: (a) clear commitment by hospital management is needed to assure resources for QI and guarantee the involvement of all relevant departments in the QI effort, (b) an approach for gathering quality data assuring inclusion of the majority of cases with sepsis is needed, and (c) structures for hospital-wide QI efforts need to be established for achieving a sustainable long-term reduction of sepsis mortality.

Using claims data for performance measurement has the advantages to cover all diagnosed cases with data being readily available and needing minimal time and costs [31]. This approach is extensively used within quality initiatives in the USA [32] and has also achieved first promising results in Germany [33].

### Objectives

The primary objective of this study is to decrease sepsis-related mortality in the participating hospitals compared to the average of all German hospitals. To obtain this objective, all hospitals regularly receive quality reports based on claims data of all in-patient cases with sepsis and implement a multifaceted intervention.

## Methods

### Context

The German Quality Network Sepsis (GQNS) was founded in February 2016. The participating hospitals committed to improve the quality of care for patients with sepsis and thereby to decrease sepsis-related hospital mortality. The start-up period of the GQNS is funded by grants from the German Federal Ministry of Education and Research (BMBF) via the integrated research and treatment Center for Sepsis Control and Care (CSCC) at the Jena University Hospital and runs from August 2015 to July 2018. The funded start-up phase and its scientific evaluation use the acronym ICOSMOS (quality Improvement in infection COntrol and Sepsis management in MOdel regionS). Additional funding is obtained via an annual fee paid by the participating hospitals. After the start-up-period, the GQNS will continue its work based on additional funding and on the fees of the participating hospitals.

**Fig. 1:**
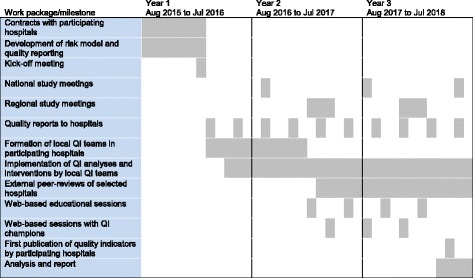
Study timeline. Study timeline and milestones are presented for the 48 hospitals initially enrolled in the German Quality Network Sepsis. A final analysis comparing these 48 intervention hospitals to all other German hospitals will be done with a delay of 1.5 years when the German national claims data will be made available by the Federal Bureau of Statistics

This study protocol describes the conduction and evaluation of the start-up-period of the GQNS. Figure 1 describes the timeline of the study. The intervention started in March 2016 with the first retrospective reporting of quality indicators including the years 2014–2015 to the participating hospitals. This study protocol takes into account the SQUIRE 2.0 recommendations [34].

#### Major principles

The GQNS fulfills the essential features of a quality collaborative [35] by being an organized multifaceted approach which (a) has the specific topic of improving quality of inpatient sepsis care, (b) involves clinical and QI experts who provide ideas and support, (c) implements QI teams in the participating hospitals, (d) uses measurable targets by sepsis-related hospital mortality, and (e) applies a series of structured interventions.

Major principles of the GQNS are as follows: (a) focusing on outcome quality by using sepsis-related hospital mortality as major quality indicator; (b) measuring quality based on claims data readily available at each participating hospital; (c) assuring validity of quality comparisons by implementing a complex risk-adjustment model; (d) aiming at hospital-wide quality improvement by formation of local multidisciplinary QI teams led by committed local champions; (e) implementing education based on Surviving Sepsis Campaign guidelines among all involved health care workers; (f) assuring full support by senior management of participating hospitals by their commitment to an annual participation fee and the publication of risk-adjusted sepsis-related hospital mortality; (g) ownership of the collaborative by the participants by establishing structures that assure participatory action; and (h) bringing the expertise available within the collaborative to action by a system of peer-reviews between the participating hospitals.

#### Project organization

The organization of the GQNS is regulated in bylaws ratified by the participating hospitals. All responsibility for the QI efforts is at the side of the individual participating hospitals. Local multidisciplinary QI teams have repeatedly been recommended for enhancing guideline implementation especially regarding sepsis [12, 19, 21, 36, 37]. Likewise, participating hospitals of the GQNS are encouraged to name local champions and establish QI teams involving all relevant departments for improving quality of sepsis care, e.g., intensive and intermediate care units, surgical and medical wards, emergency departments, and quality management departments.

The central study coordinating bureau, hosted by the CSCC, supports the QI efforts of the participating hospitals and coordinates accompanying scientific studies. Means of supporting the QI teams are as follows: (a) developing the model for risk-adjustment and specifications for quality reporting; (b) hosting the study homepage providing literature, instructions, educational material, technical and status reports, and online tutorials; (c) organizing national and regional study meetings; (d) coordinating communication and information exchange within the GQNS; (e) counseling local QI teams; (f) measuring the progress of QI efforts and barriers to change and giving feedback on these data to QI teams and senior hospital managements on a regular basis; (g) organizing online lectures and discussions with clinical experts; and (h) organizing web-based discussions with champions of successful international QI initiatives on sepsis care.

Structures of the GQNS guarantee participatory action and ownership of the collaboration by the member hospitals. Major decisions are made in the general assembly of representatives of all participating hospitals. A steering committee is elected among the members of the GQNS, to supervise and assist the work of the coordinating bureau. A committee of experts recruited from the participating hospitals organizes the peer-reviews. Claims data are collected and processed to make quality reports by the medical information technology service provider 3M Health Information Systems (3M). The GQNS is part of the Global Sepsis Alliance’s Quality Improvement Committee [38].

#### Participating hospitals

Eligible for participation in the GQNS were acute care hospitals with at least one adult intensive care unit. Invitation letters were sent to managements of hospitals which were participating in former or ongoing sepsis-related quality initiatives or research networks, to regional and national hospital groups, and to all German university hospitals. The active recruitment process started in Aug 2015 and ended in Feb 2016 and aimed at including 30 hospitals. Forty-eight hospitals were enrolled in March 2016, when the first quality reports were distributed. Characteristics of these hospitals are presented in Table 1.

**Table 1:** Characteristics of 48 initially enrolled hospitals

Characteristic	*n* (%)
Level of care	
Primary or secondary level	29 (60%)
Tertiary level	19 (40%)
Academic status	
Non-university hospital	39 (81%)
University hospital	9 (19%)
Number of beds	
≤ 400	15 (31%)
401–900	17 (35%)
901–1200	8 (17%)
> 1200	8 (17%)

Forty-eight hospitals, which were enrolled at the beginning of the intervention phase (March 2016), will be used for the evaluation of the intervention effect of the start-up period

### Interventions

The core interventions in the GQNS are (a) reporting and publication of quality indicators; (b) case analyses within the participating hospitals; (c) peer-reviews for hospitals, which are outliers in the quality reports; and (d) hospital-wide staff education in participating hospitals.

The only mandatory intervention is the reporting, benchmarking, and publication of quality indicators. All hospitals committed to provide their claims data for calculation of quality indicators and to publish their main quality indicators in comparison to the German average. The study coordination bureau provides encouragement, information, and support regarding the conduction of case analyses and staff education. It also offers peer-reviews to hospitals where quality indicators suggest low quality of care. But the responsibility for implementing case analyses and staff education is on the side of the participating hospitals and the participation in a peer-review is voluntary. Hospitals are free to develop and implement further interventions suitable to their local conditions.

#### Reporting and publication of quality indicators

There is consensus that valid and scientifically rigorous measures of performance are a means to improve health care and a central aspect of successful quality initiatives [39–42].

The primary quality indicator measuring outcome quality of sepsis care is risk-adjusted hospital mortality among cases with sepsis with organ dysfunction including septic shock. It also serves as the primary end-point of the study.

##### Measurement of risk-adjusted hospital mortality

To achieve inclusion of all cases diagnosed with sepsis and to make quality measurement also feasible for small hospitals without specialized research staff, risk-adjusted hospital mortality of cases with sepsis is measured based on claims data [31, 33]. A diagnosis-related group (DRG) system is used to structure reimbursement of hospitals by health insurances in Germany. Every hospital provides a standardized data set (DRG data) of all treated cases to the Federal Bureau for Hospital Reimbursement on a yearly base. Additionally, the German Federal Bureau of Statistics provides the anonymized DRG data of all German hospitals for scientific research with a 1.5-year delay (e.g., data of 2015 available 2017). DRG data contain demographic characteristics, International Classification of Diseases codes in its 10th version (ICD-10), codes of the German Classification of Procedures in Medicine, treating hospital departments, length of stays per department, and information regarding hospital admission and discharge.

**Fig. 2:**
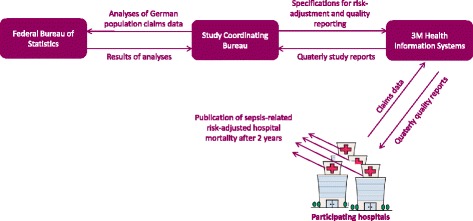
Flows of information in the German Quality Network Sepsis. The study coordinating bureau develops the risk-model based on analyzing the German population claims data hosted by the Felderal Bureau of Statistics. It further provides the specifications for calculating quality indicators to 3M Health Information Systems. Based on quarterly transmissions of claims data by the participating hospitals, 3M produces quality reports and delivers them to the hospitals. Hospitals committed to publish their sepsis-related risk-adjusted hospital mortality in comparison to the German average after 2 years of participation

Every participating hospital sends its pseudonymized DRG data to 3M on a quarterly basis (Fig. 2). Based on specifications provided by the study coordination bureau, 3M calculates quality indicators and provides quality reports to the participating hospitals. Cases with sepsis are identified based on specific ICD-10-codes and classified in subgroups according to the 1992 ACCP/SCCM sepsis definitions (Appendix) [43]. Incidence and risk-adjusted mortality is reported for cases with sepsis and organ dysfunction including septic shock as well as the subgroup of patients receiving more than 24 h of ventilation. For cases with sepsis without organ dysfunction, only incidence and observed mortality rate are reported.

Statistical methods of risk adjustment are needed to control for differences in patient-related risk-factors between hospitals and allow for valid comparisons of quality [32]. Risk-adjusted performance comparisons based on claims data have been applied to hospital profiling regarding several diseases in the USA [44–46]. The GQNS uses an external risk adjustment algorithm [47] by developing a risk model within the DRG data of all German hospitals and applying this model to the sample of hospitals within the GQNS (Fig. 2). This resembles the methodology used by the US Agency for Health Care Research and Quality Improvement (AHRQ) Inpatient Quality Indicators [48]. The risk model currently in use was developed following established methodologies [44, 49] based on all DRG-cases coded with severe sepsis in German hospitals in 2015 (manuscript in preparation). The model involves about 50 risk factors, e.g., comorbidity categories based on Charlson and Elixhauser indexes [50, 51], and achieves a predictive validity comparable to recently published models based on US American claims data [52, 53]. The risk model is applied to the hospitals in the GQNS by calculating expected mortality rates, standardized mortality ratios (SMR, “Observed over Expected Estimator”), and risk standardized mortality rates (RSMR) with their respective confidence intervals [47, 48, 54] among cases with sepsis with organ dysfunction, cases with septic shock, and cases with organ dysfunction including septic shock and ventilation of more than 24 h. The risk-model and adjustment methodology are constantly improved based on feedback by participating hospitals, research results, and yearly updates of the German hospital population DRG data.

##### Reporting of risk-adjusted mortality to hospitals

**Fig. 3:**
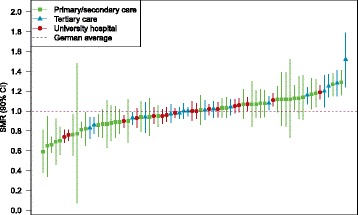
Schematic example of graphical reporting of quality indicators. Presented are SMRs (standardized mortality ratios) with 90% confidence intervals (CI) for cases with sepsis with organ dysfunction (including septic shock) in 2016. Internal quality reporting uses 90% CIs to increase sensitivity for finding true deviations from average quality. Publications of quality indicators by participating hospitals will use 95% CIs. GQNS: German Quality Network Sepsis

Quality indicators are provided to the hospitals via 3M on a quarterly base. A web-browser-based tool with numerical as well as graphical presentations is used. Incidence, observed mortality and risk-adjusted mortality within subgroups of cases with sepsis are presented for each hospital. Reports allow comparison of risk-adjusted mortality to the German average, among the hospitals of the GQNS, among hospitals of specific levels of care, and over time. Displaying confidence intervals prevents interpreting merely random differences. Figure 3 provides a schematic example of a graphical report of SMRs.

Additionally, each hospital receives a report using Microsoft Excel© including information on incidence and risk-adjusted mortality of sepsis cases within each of its departments. This report also presents lists of pseudonymized individual cases of the respective hospital including their individually predicted probability of death based on the risk model. Based on these lists, hospitals can perform in-depth single-case analyses (see below).

##### Publication of risk-adjusted mortality indicators

Public comparative reporting of health care provider performance measures is regarded a key element for improving quality of health care [55, 56]. There is evidence that public reporting has greater effects on organizational improvement efforts than private disclosure of the same data alone [57–60]. Senior managements of hospitals within the GQNS consented to publish their hospitals’ risk-adjusted mortality indicators compared to the average of the German DRG data after 2 years of participation. By doing this, hospitals document their commitment to quality improvement, accountability for quality of sepsis care, and commitment to principles of transparency in health care. Figure 2 presents the system of quality measurement and reporting within the GQNS.

#### Case analyses

Analysis of individual case records can reveal preventable deaths as well as underlying shortcomings in care [61–63]. Analysis of adverse events among cases with sepsis has revealed that problems in guideline adherence regarding diagnosis and treatment, like delayed recognition of deterioration, delayed source control, and inadequate fluid and antimicrobial therapy, but also structural problem like hand-over practices from normal ward to ICU cause preventable deaths [3, 4].

Within the GQNS, individual probabilities for death are predicted based on the risk model and provided to the participating hospitals. The study coordination bureau provided information on how to conduct case analyses. Deceased cases with a predicted low risk of in-hospital mortality are used to identify possible problems in the quality of care or ICD-10 coding. Hospitals can depseudonymize these cases and analyze individual medical records using established methods [62, 64, 65]. Interdisciplinary case discussions moderated by members of the local QI teams can be used to consent on goals for quality improvement.

#### Peer-reviews

Peer-review, a process by which physicians evaluate each other’s performance, has a long tradition in efforts to improve health care quality [66]. A system of peer-reviews using external peers from other hospitals is used within a quality initiative involving 400 hospitals (Initiative Qualitätsmedizin [IQM]) [67]. It has been shown to be able to detect areas of possible improvement of care and to be related to decreases in mortality in diseases like heart failure and pneumonia [33]. Based on this methodology, a peer-review committee consisting of specifically trained clinicians is set up within the GQNS and working instructions have been defined. An external peer-review is suggested to hospitals with SMRs for patients with sepsis with organ dysfunction including shock substantially higher than the German average. Additionally, hospitals can call for a peer-review by themselves. External peers investigate a sample of the retrospective case records. These records are identified among cases that died despite of a low predicted risk. Based on case-analysis, external peers will discuss improvement strategies with involved local departments.

#### Staff education

The main focus of staff education is the implementation of strategies for increasing awareness and early recognition of sepsis as well as the implementation of key elements of the updated Surviving Sepsis Campaign guidelines among physician and nurses, as well as all other health care workers involved in care for patients with sepsis [8, 68, 69]. The content of the education is focused on a small number of essentials (“Sepsis Bundles”). Education is implemented by the local QI teams. The study coordination bureau supports the local QI teams by providing educative material (presentations, pocket cards, posters) developed during the MEDUSA trial. Furthermore, online lectures and discussions involving QI improvement experts are provided.

#### Further interventions

Participating hospitals will be encouraged to implement a protocol for early recognition of sepsis in the emergency department, recognition of deteriorating patients or patients at risk on regular wards (standardized screening for sepsis), and to establish rapid response teams, which are mandatory or widely implemented in some countries but not in Germany [70]. Furthermore, hospitals will be encouraged to get active in at least one component of the national hospital surveillance program (Krankenhaus-Infektions-Surveillance-System KISS) (http://www.nrz-hygiene.de/en/surveillance/hospital-infection-surveillance-system/).

### Study of the interventions

The effect of participating in the start-up period of the GQNS will be evaluated by comparing the participating hospitals to all other German hospitals regarding the difference between the retrospective baseline period from January 2014 to December 2015 to the intervention period from April 2016 to March 2018 (quasi-experimental difference-in-differences design) [71]. This approach is more valid than uncontrolled before-after comparisons that were used in previous quality initiatives on sepsis [23], since it is able to distinguish the intervention effect from secular changes that would have occurred without the intervention [71]. Beside secular changes, the so called Will Rogers phenomenon might bias changes in observed mortality across time [71, 72]. This phenomenon occurs if an intervention increases awareness for a disease, which results in increased detection and coding also of less severely ill cases. By incorporating an adjustment for risk factors in the difference-in-differences comparison, the analyses will try to correct for this bias. The analyses will evaluate the overall effect of participating in the GQNS, but they will not be able to distinguish which intervention elements cause possible changes.

#### Measures for studying processes and implementation of the interventions

The study coordination bureau of the GQNS receives a study report by 3M on a quarterly base that informs about the quality indicators of each participating hospital as well as about hospitals that failed to provide their claims data. Hospitals that do not provide their claims data despite several reminders are excluded from the quality collaborative. To assess fidelity and dose of the local implementation of interventions in the participating hospitals, the coordination bureau of the GQNS regularly surveys local QI team leaders by standardized online questionnaires. These questionnaires contain items regarding departments participating in the QI teams, extend of interdisciplinary analyses of quality indicators, extend of interdisciplinary case analyses, extend of education of staff, and implementation of further interventions like existence and scope of rapid response teams. The questionnaire also contains items regarding barriers to change like lacking resources or support by management, as well as items that evaluate the quality of tools and support provided by the coordination bureau. The questionnaire was developed based on results of qualitative interviews conducted during the MEDUSA trial [30]. Peer-review visits are documented and evaluated using paper-pencil surveys using standardized questionnaires both by the external peers and the involved local clinicians. These questionnaires were adapted from existing tools used in other German quality initiatives [67, 73].

#### Outcome measures

Hospital mortality of cases with sepsis with organ dysfunction (including septic shock) in the participating hospitals is the primary end-point of the study, since the newly revised consensus definitions of sepsis and septic shock (“Sepsis-3”) exclude cases without organ dysfunction [2]. Since the evaluation of the study effect relies on claims data, it is not possible to fully assess and guarantee completeness and accuracy of data. To allow hospitals to identify possible under-coding of cases with sepsis the incidence of sepsis, severe sepsis and septic shock is presented and compared between hospitals in the quality reports.

### Analyses

#### Analysis of the primary outcome

The final analysis of the intervention effect will be conducted using German national DRG data. The change in hospital mortality between the baseline period (January 2014 to December 2015) and the intervention period (April 2016 to March 2018) will be compared between the 48 hospitals initially enrolled in the GQNS and control hospitals. These are all non-participating German hospitals, whose DRG data are also available from the Federal Bureau of Statistics. Hospitals entering the GQNS after March 2016 will be excluded from this comparison. This comparison will be conducted in 2020 when the national DRG data for 2018 will be available from the Federal Bureau of Statistics. The primary analysis will evaluate the difference-in-differences in a hierarchical generalized linear model controlling for clustering of cases in hospitals by testing the statistical interaction between period (baseline vs. intervention) and group (48 hospitals enrolled in GQNS vs. control hospitals). Patient-related risk-factors will be considered as covariates. As a secondary analysis, the trajectories of change across time before and after intervention will be compared between intervention and control hospitals by a piecewise hierarchical model [74].

A preliminary evaluation of the study effect will be performed after completion of the intervention period in March 2018 but only relying on the data of the 48 intervention hospitals (before-after-comparison). A secondary preliminary analysis will be done by a piecewise hierarchical model to investigate the change in the trajectory of hospital mortality over time between periods.

#### Sensitivity analyses and planned subgroup analyses

Differences across time between hospitals of the GQNS and control hospitals might result from changes in coding in the intervention hospitals, e.g., improved ICD-coding of sepsis or comorbidities. Therefore, incidences of sepsis without organ dysfunction, sepsis with organ dysfunction and septic shock as well as incidences of mortality risk-factors will be compared across time between intervention and control hospitals. Analyses of the primary outcome will be repeated within these three subgroups of sepsis cases.

Additionally, hospitals will be categorized based on the degree of their QI efforts during the intervention period but without inspection of their primary outcome. The categorization will be based on the results of the regularly conducted surveys of local QI team leaders. The analyses of the primary end-point will be repeated within the subgroups of hospitals with high QI effort and hospitals with low QI effort.

#### Sample size considerations

Concerning the baseline period (January 2014 to December 2015), 45,419 cases with sepsis were included among the initially enrolled 48 hospitals, of which 27,331 (60%) were diagnosed with sepsis with organ dysfunction (including septic shock), resulting in approximately 1000 cases per hospital across both the baseline and intervention period. Comparing the intervention hospitals with all other German hospitals across a baseline and intervention period resembles a controlled before-after trial on the cluster level. Therefore, our sample size calculation used a design factor developed for cluster-randomized before-after trials [75]. We observed a baseline mortality of 44% and an interclass correlation (ICC) of 0.02 among the included 48 hospitals. Based on the results of the Surviving Sepsis Campaign [15], a reduction in mortality to 40% was expected for the 2-year intervention period. A Fisher’s exact test comparing intervention and control conducted at α-level 0.05 with a power of 0.9 would require a total sample size of 6476. Correcting this sample size by the obtained design factor of 3.75, a total of 26 hospitals (13 control and 13 intervention) each including 1000 cases would be necessary in a cluster randomized before-after trial. Given 48 intervention hospitals and more than 1000 control hospitals in the national German DRG data, our study should be adequately powered to detect the expected intervention effect.

### Ethical considerations

The study underwent review by the internal review board of the Jena University Hospital (IRB protocol 4536-08/15) and the data protection supervisor of the Free State of Thuringia, Germany. Since individual case data are only obtained in the form of pseudonymized claims data, the need for informed consent by patients was waived. Quality reports present only aggregated data by hospital. Hospital names are pseudonymized, and each hospital knows only its own pseudonym. Only 3M and the study coordination bureau can de-pseudonymize all hospitals. Individual case data are only reported to the hospital where the cases were treated. For all kinds of surveys of QI team members, hospital staff, or peer-reviewers, participants will be informed about aims of the surveys and methods for protection of data privacy. Informed consent will be implied by participation in the survey, survey data will be stored in pseudonymized or anonymized form only, and all public reports of survey data will contain only aggregated data allowing no identification of individual participants.

### Project status

The GQNS was officially started with a kick-off meeting in February 2016. The first quality reports were distributed in March 2016 to the initially enrolled 48 hospitals. Till October 2017, quality reports have been distributed five times. Based on the public awareness of the GQNS, additional hospitals entered the network after the active recruitment. In September 2017, the number of participating hospitals reached 75, clearly surpassing the initially planned number. Participating hospitals are located all over Germany and include hospitals of all levels of care, among them 18 university hospitals (of 39 in Germany). In August 2016 and August 2017, two surveys of local QI team leaders were performed to assess the current progress and barriers to change in the participating hospitals and results were reported to senior hospital managements. Between March 2017 and September 2017, the first four online lectures and between May 2017 and September 2017 the first three external peer-reviews took place.

## Discussion

The goal of this project is to establish structures for quality improvement of care for patients with sepsis and thereby to decrease sepsis-related in-hospital mortality. The GQNS is the first quality collaborative using claims data and a complex risk-adjustment to measure and improve quality of care for patients with sepsis. Because of this pragmatic approach 75 hospitals signed contracts for participation.

Previous QI initiatives to improve sepsis care relied on gathering prospective clinical data in an uncontrolled before-and-after design [15, 17–21]. Based on these studies, it is impossible to distinguish the intervention effects from general secular trends [71]. The first cluster-randomized trial which set out to test for effects of elements of a QI collaborative on sepsis-related mortality (MEDUSA trial) failed partly because of incomplete and delayed inclusion of cases with sepsis. Our study design overcomes both of these shortcomings. Using claims data makes all patients with coded sepsis available for analysis. Based on a German national claims database, it is possible to evaluate the effect of the QI initiative in a difference-in-differences design comparing intervention hospitals to all other German hospitals.

The approach of our study of course has some limitations. First, under- or over-coding of sepsis might occur in claims data [76]. However, for every hospital, the degree of under-documentation has decreased considerably, as since 2007 appropriate coding of sepsis cases has become a financial interest for the hospitals. On the other hand, over-coding of sepsis cases for financial reasons is systematically controlled for by the Medical Review Board of the Statutory Health Insurance Funds. A second limitation is the time-lag of 1.5 years until German national claims data are available for analysis delaying the final analysis of the intervention effect. A preliminary evaluation of the effect can only rely on data of the intervention hospitals. Finally, quality improvement in sepsis care has been described to be a marathon and not a short sprint [77]. The 2-year intervention period of the start-up phase of the GQNS might be too short for attaining substantial effects on hospital mortality in a large sample of hospitals. The aim of this start-up period is to build a long-lasting quality initiative and to establish structures for sustained quality improvement. A reevaluation of the effects of participating in the GQNS after a longer period of time—comparable to the duration of other successful large-scale QI initiatives [15]—might be necessary.

If this study finds that the approach of using claims data in combination with a complex risk-adjustment is useful for measuring and improving quality of sepsis care, the approach may also be applied to other diseases.

## Appendix

### Definition of cases with sepsis by German ICD-10 codes

Sepsis without organ dysfunction: A40.0, A40.1, A40.2, A40.3, A40.8, A40.9, A41.0, A41.1, A41.2, A41.3, A41.4, A415.1, A41.52, A41.58, A41.8, A41.9, R65.0.

Sepsis with organ dysfunction but without septic shock: R65.1.

Septic shock: R57.2.

## Abbreviations

3M: 3M Health Information Systems
CSCC: Center for Sepsis Control and Care
DRG: Diagnostic related groups
GQNS: German Quality Network Sepsis
MEDUSA trial: Medical EDUcation for sepsis Source control and Antibiotics, ClinicalTrials.gov identifier NCT01187134
QI: Quality improvement
RSMR: Risk standardized mortality rate
SMR: Standardized mortality ratio

## References

[1] Angus DC, van der Poll T (2013). Severe sepsis and septic shock. N Engl J Med.

[2] Singer M, Deutschman CS, Seymour CW (2016). The third international consensus definitions for sepsis and septic shock (sepsis-3). JAMA-J Am Med Assoc.

[3] Clinical Excellence Commission. Recognition and management of sepsis. 2012. http://www.cec.health.nsw.gov.au/__data/assets/pdf_file/0004/259375/patient-safety-report-sepsis-2012.pdf. Accessed 15 Jan 2017.

[4] Goodwin APL, Srivastava V, Shotton H, et al. Just say sepsis! A review of the process of care received by patients with sepsis; 2015. http://www.ncepod.org.uk/2015report2/downloads/JustSaySepsis_FullReport.pdf. Accessed 15 Jan 2017.

[5] Fleischmann C, Scherag A, Adhikari NKJ (2016). Assessment of global incidence and mortality of hospital-treated sepsis. Am J Respir Crit Care Med.

[6] Torio CM, Moore BJ (2016). National inpatient hospital costs: the most expensive conditions by payer, 2013. Internet.

[7] World Health Assembly Executive Board. EB140.R5 Improving the prevention, diagnosis and management of sepsis. 2017 [PDF]. http://apps.who.int/gb/ebwha/pdf_files/EB140/B140_R5-en.pdf. Accessed 26 Oct 2017.

[8] Dellinger RP, Levy MM, Rhodes A (2013). Surviving Sepsis Campaign: international guidelines for management of severe sepsis and septic shock, 2012. Intensive Care Med.

[9] Ferrer R, Artigas A, Suarez D (2009). Effectiveness of treatments for severe sepsis a prospective, multicenter, observational study. Am J Respir Crit Care Med.

[10] Ferrer R, Martin-Loeches I, Phillips G (2014). Empiric antibiotic treatment reduces mortality in severe sepsis and septic shock from the first hour: results from a guideline-based performance improvement program. Crit Care Med.

[11] Gaieski DF, Mikkelsen ME, Band RA (2010). Impact of time to antibiotics on survival in patients with severe sepsis or septic shock in whom early goal-directed therapy was initiated in the emergency department. Crit Care Med.

[12] Bloos F, Thomas-Ruddel D, Ruddel H (2014). Impact of compliance with infection management guidelines on outcome in patients with severe sepsis: a prospective observational multi-center study. Crit Care.

[13] Kumar A, Roberts D, Wood KE (2006). Duration of hypotension before initiation of effective antimicrobial therapy is the critical determinant of survival in human septic shock. Crit Care Med.

[14] Levy MM, Artigas A, Phillips GS, et al. Outcomes of the surviving sepsis campaign in intensive care units in the USA and Europe: a prospective cohort study. Lancet Infect Dis. 2012;12(12):919-24. 10.1016/S1473-3099(12)70239-6 23103175

[15] Levy MM, Rhodes A, Phillips GS (2015). Surviving Sepsis Campaign: association between performance metrics and outcomes in a 7.5-year study. Crit Care Med.

[16] Seymour CW, Gesten F, Prescott HC (2017). Time to treatment and mortality during mandated emergency care for sepsis. N Engl J Med.

[17] Burrell AR, McLaws ML, Fullick M (2016). SEPSIS KILLS: early intervention saves lives. Med J Aust.

[18] Miller RR, Dong L, Nelson NC (2013). Multicenter implementation of a severe sepsis and septic shock treatment bundle. Am J Respir Crit Care Med.

[19] Ferrer R, Artigas A, Levy MM (2008). Improvement in process of care and outcome after a multicenter severe sepsis educational program in Spain. JAMA-J Am Med Assoc.

[20] Castellanos-Ortega A, Suberviola B, Garcia-Astudillo LA (2010). Impact of the Surviving Sepsis Campaign protocols on hospital length of stay and mortality in septic shock patients: results of a three-year follow-up quasi-experimental study. Crit Care Med.

[21] Noritomi DT, Ranzani OT, Monteiro MB (2014). Implementation of a multifaceted sepsis education program in an emerging country setting: clinical outcomes and cost-effectiveness in a long-term follow-up study. Intensive Care Med.

[22] Scheer CS, Fuchs C, Kuhn S-O, et al. Quality improvement initiative for severe sepsis and septic shock reduces 90-day mortality: a 7.5-year observational study. Crit Care Med. 2016; Publish Ahead of Print 10.1097/CCM.0000000000002069 27661863

[23] Damiani E, Donati A, Serafini G (2015). Effect of performance improvement programs on compliance with sepsis bundles and mortality: a systematic review and meta-analysis of observational studies. PLoS One.

[24] Fleischmann C, Thomas–Rueddel DO, Hartmann M, et al. Fallzahlen und Sterblichkeitsraten von Sepsis-Patienten im Krankenhaus. Dtsch Arztebl Int 2016;113(10):159-166. 10.3238/arztebl.2016.0159 PMC4814768 27010950

[25] Kaukonen KM, Bailey M, Suzuki S (2014). Mortality related to severe sepsis and septic shock among critically III patients in Australia and New Zealand, 2000-2012. JAMA-J Am Med Assoc.

[26] Shankar-Hari M, Harrison DA, Rowan KM. Differences in impact of definitional elements on mortality precludes international comparisons of sepsis epidemiology—a cohort study illustrating the need for standardized reporting. Crit Care Med. 2016; Publish Ahead of Print 10.1097/CCM.0000000000001876 27352126

[27] Lagu T, Rothberg MB, Shieh MS (2012). Hospitalizations, costs, and outcomes of severe sepsis in the United States 2003 to 2007. Crit Care Med.

[28] Engel C, Brunkhorst FM, Bone HG (2007). Epidemiology of sepsis in Germany: results from a national prospective multicenter study. Intensive Care Med.

[29] Bloos F, Rüddel H, Thomas-Rüddel D (2017). Effect of a multifaceted educational intervention for anti-infectious measures on sepsis mortality: a cluster randomized trial. Intensive Care Med.

[30] Matthaeus-Kraemer CT, Thomas-Rueddel DO, Schwarzkopf D (2015). Barriers and supportive conditions to improve quality of care for critically ill patients: a team approach to quality improvement. J Crit Care.

[31] Iezzoni LI (1997). Assessing quality using administrative data. Ann Intern Med.

[32] Iezzoni LI, Smith PC, Mossialos E, Papanicolas I, Leatherman S (2009). Risk adjustment. Performance measurement for health system improvement: experiences, challenges and prospects.

[33] Nimptsch U, Mansky T (2013). Quality measurement combined with peer review improved German in-hospital mortality rates for four diseases. Health Aff (Millwood).

[34] Ogrinc G, Davies L, Goodman D, et al. SQUIRE 2.0 (Standards for QUality Improvement Reporting Excellence): revised publication guidelines from a detailed consensus process. BMJ Qual Saf. 2015; 10.1136/bmjqs-2015-004411 PMC5256233 26369893

[35] Schouten LMT, Hulscher M, van Everdingen JJE (2008). Evidence for the impact of quality improvement collaboratives: systematic review. Br Med J.

[36] Schouten LM, Hulscher ME, van Everdingen JJ (2008). Evidence for the impact of quality improvement collaboratives: systematic review. BMJ.

[37] Hulscher M, Schouten LMT, Grol R (2013). Determinants of success of quality improvement collaboratives: what does the literature show?. BMJ Qual Saf.

[38] Global Sepsis Alliance Quality Improvement Committee. https://www.global-sepsis-alliance.org/qic/. Assessed 17 Oct 2017.

[39] Brook RH, McGlynn EA, Cleary PD (1996). Quality of health care .2. Measuring quality of care. N Engl J Med.

[40] Duckers MLA, Spreeuwenberg P, Wagner C (2009). Exploring the black box of quality improvement collaboratives: modelling relations between conditions, applied changes and outcomes. Implement Sci.

[41] Ovretveit J, Gustafson D (2002). Evaluation of quality improvement programmes. Qual Saf Health Care.

[42] Kaplan HC, Brady PW, Dritz MC (2010). The influence of context on quality improvement success in health care: a systematic review of the literature. Milbank Q.

[43] Bone RC, Balk RA, Cerra FB (1992). American College of Chest Physicians/Society of Critical Care Medicine consensus conference: definitions for sepsis and organ failure and guidelines for use of innovative therapies in sepsis. Crit Care Med.

[44] Krumholz HM, Wang Y, Mattera JA (2006). An administrative claims model suitable for profiling hospital performance based on 30-day mortality rates among patients with an acute myocardial infarction. Circulation.

[45] Krumholz HM, Lin Z, Drye EE (2011). An administrative claims measure suitable for profiling hospital performance based on 30-day all-cause readmission rates among patients with acute myocardial infarction. Circ Cardiovasc Qual Outcomes.

[46] Bratzler DW, Normand SLT, Wang Y, et al. An administrative claims model for profiling hospital 30-day mortality rates for pneumonia patients. PLoS One. 2011;6(4) 10.1371/journal.pone.0017401 PMC3075250 21532758

[47] DeLong ER, Peterson ED, DeLong DM (1997). Comparing risk-adjustment methods for provider profiling. Stat Med.

[48] Agency for Health Care Research and Quality Improvement. Quality indicator emprical methods—revised November 2014. 2015 [PDF]. http://www.qualityindicators.ahrq.gov/Downloads/Resources/Publications/2015/Empirical_Methods_2015.pdf. Accessed 13 Oct 2017.

[49] Krumholz HM, Brindis RG, Brush JE (2006). Standards for statistical models used for public reporting of health outcomes—an American Heart Association scientific statement from the quality of care and outcomes research interdisciplinary writing group—cosponsored by the council on epidemiology and prevention and the stroke council—endorsed by the American College of Cardiology Foundation. Circulation.

[50] Charlson ME, Pompei P, Ales KL (1987). A new method of classifying prognostic co-morbidity in longitudinal studies—development and validation. J Chronic Dis.

[51] Elixhauser A, Steiner C, Harris DR (1998). Comorbidity measures for use with administrative data. Med Care.

[52] Thomas-Rüddel D, Schwarzkopf D, Fleischmann C, et al. Development of a sepsis mortality prediction model for use with German hospital claims data. Intensive Care Med Exp. 2016;4(1) ESICM LIVES 2016: part two-Abstract A2504

[53] Ford DW, Goodwin AJ, Simpson AN (2016). A severe sepsis mortality prediction model and score for use with administrative data. Crit Care Med.

[54] Hosmer DW, Lemeshow S (1995). Confidence-interval estimates of an index of quality performance based on logistic regression models. Stat Med.

[55] Christianson JB, Volmar KM, Alexander J (2010). A report card on provider report cards: current status of the health care transparency movement. J Gen Intern Med.

[56] Emmert M, Hessemer S, Meszmer N (2014). Do German hospital report cards have the potential to improve the quality of care?. Health Policy.

[57] Fung CH, Lim YW, Mattke S (2008). Systematic review: the evidence that publishing patient care performance data improves quality of care. Ann Intern Med.

[58] Hafner JM, Williams SC, Koss RG (2011). The perceived impact of public reporting hospital performance data: interviews with hospital staff. Int J Qual Health Care.

[59] Hibbard JH, Stockard J, Tusler M (2005). Hospital performance reports: impact on quality, market share, and reputation. Health Aff (Millwood).

[60] Contandriopoulos D, Champagne F, Denis JL (2014). The multiple causal pathways between performance measures’ use and effects. Med Care Res Rev.

[61] Hogan H, Healey F, Neale G (2012). Preventable deaths due to problems in care in English acute hospitals: a retrospective case record review study. BMJ Qual Saf.

[62] Hogan H, Healey F, Neale G (2014). Learning from preventable deaths: exploring case record reviewers’ narratives using change analysis. J R Soc Med.

[63] Nguyen HB, Lynch EL, Mou JA (2007). The utility of a quality improvement bundle in bridging the gap between research and standard care in the management of severe sepsis and septic shock in the emergency department. Acad Emerg Med.

[64] Wu AW, Lipshutz AM, Pronovost PJ (2008). EFfectiveness and efficiency of root cause analysis in medicine. JAMA.

[65] Pronovost PJ, Holzmueller CG, Martinez E (2006). A practical tool to learn from defects in patient care. Joint Comm J Qual Patient Saf.

[66] Edwards MT (2013). A longitudinal study of clinical peer review’s impact on quality and safety in US hospitals. J Healthc Manag.

[67] Rink O. Wie wir Qualität verbessern. In: Martin J, Rink O, Zacher J, editors. : Handbuch IQM; 2014.

[68] Lilly CM (2014). The ProCESS trial—a new era of sepsis management. N Engl J Med.

[69] Yealy DM, Kellum JA, Huang DT (2014). A randomized trial of protocol-based care for early septic shock. N Engl J Med.

[70] Chen J, Ou L, Flabouris A (2016). Impact of a standardized rapid response system on outcomes in a large healthcare jurisdiction. Resuscitation.

[71] Iwashyna TJ, Angus DC (2014). Declining case fatality rates for severe sepsis good data bring good news with ambiguous implications. JAMA-J Am Med Assoc.

[72] Feinstein AR, Sosin DM, Wells CK (1985). The will Rogers phenomenon. N Engl J Med.

[73] Felsenstein M (2011). Peer Review: Lernen auf Gegenseitigkeit. Dtsch Arztebl Int.

[74] Gerber JS, Prasad PA, Fiks AG (2013). Effect of an outpatient antimicrobial stewardship intervention on broad-spectrum antibiotic prescribing by primary care pediatricians a randomized trial. JAMA-J Am Med Assoc.

[75] Hemming K, Taljaard M (2016). Sample size calculations for stepped wedge and cluster randomised trials: a unified approach. J Clin Epidemiol.

[76] Jolley RJ, Sawka KJ, Yergens DW (2015). Validity of administrative data in recording sepsis: a systematic review. Crit Care.

[77] Bhardwaj A, Mikkelsen ME (2017). Sepsis quality improvement initiatives: prepare for the marathon, not the sprint. Crit Care Med.

